# The Relationship Among Body Composition and Anaerobic Capacity and the Sport Level of Elite Male Motorcycle Speedway Riders

**DOI:** 10.3389/fphys.2022.812958

**Published:** 2022-04-13

**Authors:** Kamil Michalik, Stefan Szczepan, Maciej Markowski, Marek Zatoń

**Affiliations:** ^1^ Department of Human Motor Skills, Faculty of Physical Education and Sports, Wroclaw University of Health and Sport Sciences, Wroclaw, Poland; ^2^ Department of Swimming, Faculty of Physical Education and Sport, Wroclaw University of Health and Sport Sciences, Wroclaw, Poland; ^3^ Graduate Student, Faculty of Physical Education and Sport, Wroclaw University of Health and Sport Sciences, Wroclaw, Poland; ^4^ Department of Physiology and Biochemistry, Faculty of Physical Education and Sport, Wroclaw University of Health and Sport Sciences, Wroclaw, Poland

**Keywords:** motorcycle speedway racing, anaerobic performance, Wingate test, body composition, anthropometry

## Abstract

The main objective of this study was to investigate the relationship among anaerobic capacity, body composition, and sport level of male junior and senior speedway riders. Sixty riders of professional clubs in the Polish top motorcycle speedway league participated in this study. They were divided into two equal groups (*n* = 30): junior (age = 19.7 ± 1.1 years) and senior (age = 29.7 ± 5.2). Body composition assessment, Wingate test (WAnT) on cycloergometer, with analysis of acute cardiorespiratory and biochemical responses were performed. Sport level was defined as the number of heats (races) won, winning percentage, total points scored during the season, and average points scored per heat. Seniors had higher point ratings indicating sport levels. As compared to the seniors, the juniors had lower BMI 4% (*p* < 0.01) and fat tissue mass by 20.5% (*p* < 0.01). A higher power decrease of 2.3% (*p* < 0.05) in the WAnT test was found in seniors. Body height negatively correlated with all indicators of seniors’ sport level (r = −0.41 to −0.55). Peak power output negatively correlated with seniors’ sport level (r = −0.39 to −0.41). Among the seniors, there was a negative correlation between post-exercise hydrogen ion concentration (r = −0.38), carbon dioxide partial pressure (r = −0.45) and average points scored per season. Conclusion: The anthropometric characteristics of body height, lean body mass and body surface area, are significantly correlated with the sport levels of the seniors motorcycle speedway riders. When selecting motorcycle speedway riders, use of these anthropometric characteristics may aid in determining the riders with the most potential to be successful. Metabolic acidosis tolerance and gas exchange efficiency show significance in seniors, indicating the need for intense exercise sessions.

## Introduction

A high level of physical fitness is a prerequisite for effective participation in motorsport training and competitions, leading to the development of exercise adaptation, increasing tolerance to overload resulting from physical work and changes in internal homeostasis. Additionally, attention must be given to the recovery process, as well as the maintenance and regulation of body mass and composition. This is particularly important in sports where proper body composition is important for sports performance (Slater et al., 2005; Slater et al., 2014). In motorsports, such as speedway riding, it has been shown that the lower combined mass of the motorcycle speedway rider and equipment reduces the rider’s moment of inertia and inertial load, which, when generating the same level of power, has a positive effect on the acceleration phase (Martin et al., 2019). A rider with a lighter and smaller body can achieve greater acceleration and top speed (Sánchez-Muñoz et al., 2011).

Previous original and review papers on motorsports have focused on the analysis of anthropometric parameters, anaerobic and aerobic capacity (VO_2_peak) (Bach et al., 2015), cardiorespiratory and neuromuscular responses (Konttinen et al., 2008), lactate concentration and heart rate (D'Artibale et al., 2008), kinesthetic sensing (Szczepan et al., 2020), physical fitness and selected health attributes (Burr et al., 2010), as well as human performance in a broad sense (D'Artibale et al., 2018a). It has been established that in achieving a high level in motorsports, particular importance is attributed to the mechanical properties of the equipment, technical competence, physical characteristics including relatively low height and weight, adequate level of strength, balance and flexibility (Baur et al., 2005; Bach et al., 2015; D'Artibale et al., 2018a). Motorsport also requires the rider to be able to generate high upper extremity force (Baur et al., 2005) and physiological adaptation to changes induced by physical work (much of it isometric) during racing (Ascensão et al., 2008). Isometric and/or eccentric muscle contractions are necessary to absorb shocks caused by variations in terrain grip and motorcycle handling on rough terrain with sharp turns (Ascensão et al., 2008). The research cited in this section of the paper mainly refer to recreational motorcyclists, as well as professional motocross (MX), enduro, and road racing (MotoGP) competitors, all of which have different characteristics compared to speedway racing.

Motorcycle speedway racing, as a sport, has been known for over 90 years and continues to grow in popularity. League competitions in Poland are considered to be the best in the world and attract outstanding competitors from many countries. League riders can be classified in the age category of juniors (youth) <21 years of age, and seniors. The training system assumes recruitment of children, starting from 6 years of age, to mini speedway schools (participation in competitions for 8–10-year-olds) in the 50cc class. From the age of 8, the rider competes in the 125cc class (participation in competitions for 10–12-year-olds). The 250cc class includes 11-year-olds (competing at the age of 13–14). Recruitment for 500cc speedway schools starts from the age of 14 (youth competitions from 15, and the possibility of participation in the U24 Ekstraliga—U24 Elite League). Unlimited 500cc motorcycle competitions are open to those over the age of 16.

The indicator of individual riders’ sport level classification in Polish speedway leagues is the Average Heat Score (AHS) which is the total number of points scored divided by the number of heats in the match. In traditional scoring, the AHS can be from 0.00 to 3.00 (3 points are awarded for winning the heat, 2 points for 2^nd^ place, 1 point for 3^rd^ place and no points for the last place). Each rider’s AHS is updated every competition and is based on their performance in each competition (PZM—Polish Motorsport Association, 2021). Every league in the world has the same method of calculating the AHS, except for the British league, which has what is known as the CMA (Calculated Match Average). The British CMA is the averaged points per match of a given competitor (total points divided by total heats, multiplied by 4). It indicates the rider’s class and current disposition, as the CMA is updated on the 1st and 15th of each month (SCBGB—Speedway Control Bureau GB, 2021).

Competition takes place with standardized rules for all riders (FIM—Federation International Motorsport, 2020). During the heat, four competitors must complete four laps of the track (length: about 260–425 m) in a counter-clockwise direction, which takes about 60 s. The starting field (1–4) indicates the place on the track from which the competitor starts the heat. All speedway heats have a stationary start in the middle of one of the straightaways. A speedway match in Polish league consists of 15 heats and takes from two to three h.

A motorcycle weighing at least 77 kg, with a 500cc engine, one gear (no. gearbox) and no brakes. It accelerates to a speed of over one hundred kilometers per hour (FIM, 2020), while reaching 80 km per hour in about 2.4 s. The significant acceleration and maintenance of maximum speed over distance while going on the straightaways and curves places significant physical and psychological stresses on speedway riders, similar to other motorsports (D'Artibale et al., 2018b).

Interestingly, despite the enormous popularity of motorcycle speedway sport, the scientific literature on the sport is limited. A recent paper by Martin et al. (2019) analyzed the physical profile of experienced and inexperienced speedway riders based on anthropometric measurements (the lengths of extremities). Numerous motor skills tests were also used, i.e., the lower quadrant y-balance test, the upper quadrant y-balance test, the functional movement screen, isometric grip strength and isometric leg extension force. The authors concluded that low height and body mass, as well as dynamic lower extremity balance are key physical characteristics of the competitive motorcycle speedway rider. Furthermore, motorcycle speedway riders have greater hand grip strength and knee extension on the right side than on the left, and riders with higher sport levels have demonstrated significantly greater isometric strength and performance on functional tests (Martin et al., 2019).

In addition to the aforementioned strength, it seems that anaerobic capacity should demonstrate great importance in motorcycle speedway riding, due to the need for short and very intense physical activity. It appears that the Wingate test can be used to monitor the maximum power-generating ability of motorcycle speedway riders under laboratory conditions. The timing and nature of muscle work during the approximately 60-s speedway heat indicates that energy is provided by the combined interaction of aerobic and anaerobic energy pathways (Gastin, 2001). During the training sessions, lactate concentrations of >12 mmol·L^−1^ were measured after completing four laps at maximum intensity (unpublished data). The demanding and precise muscle activity during a heat occurs with increasing levels of peripheral fatigue, which affects performance (Katakura et al., 2011). Because of that, the rider must have the ability to tolerate changes in homeostasis and buffer increasing acidification to minimize fatigue (D'Artibale et al., 2008).

Because there is limited information on the importance of body composition, anaerobic capacity, cardiorespiratory and biochemical responses in elite speedway riders, this research focused on the relationships among these characteristics and sport levels in these riders. The purpose of this study had two aspects. The first aspect was to compare between juniors and seniors in terms of the body composition components, anaerobic capacity and cardiorespiratory responses achieved in the Wingate test, and the sport level, defined by total points scored, and derivatives among the competitors of the highest competition class in Poland. The second objective was to determine the relationship among the foregoing characteristics. We hypothesized that there would be no differences among body composition, anaerobic capacity, and cardiorespiratory and biochemical responses, and that juniors and seniors would differ in sport levels indicators. Furthermore, we hypothesized that high levels of anaerobic capacity and low fat mass (% FM) would be associated with sport level in both age categories.

## Materials and Methods

### Participants

The study involved 60 male motorcycle speedway riders from several clubs of the highest speedway competition class in Poland (Speedway Ekstraliga—“Speedway Elite League”). There were Polish national representatives in international events among the study participants. The subjects’ mean age, body height, body mass, systolic blood pressure, diastolic blood pressure, hemoglobin level and hematocrit are shown in Table 1. The subjects were presented as an entirety and divided into two age groups (juniors and seniors). A junior (or youth) competitor is a rider under the age of 21. The following study inclusion criteria were used: 1) contracted in Polish Speedway Ekstraliga club, 2) minimum 1 season of experience in motorcycle speedway competitions 3) no contraindications to exercise tests, 4) age above 16 years. Participants were informed of the potential risks of the study and all gave written informed consent to participate. Respondents were also informed that they could withdraw at any time. There were no dropouts in terms of participation. The study was performed in the Exercise Testing Laboratory in accordance with the Declaration of Helsinki (certified with PN-EN ISO 9001: 2001). The study was approved by the University Research Ethics Committee (6/2015).

**TABLE 1:** Participants’ characteristics—overall and both groups.

Variable	Overall (*n* = 60)		Junior (*n* = 30)		Senior (*n* = 30)	
	$x^{¯}$ ± SD	95% CI	$x^{¯}$ ± SD	95% CI	$x^{¯}$ ± SD	95% CI
Age (years)	24.7 ± 6.3	23.1–26.3	19.7 ± 1.1*	19.3–20.1	29.7 ± 5.2	27.7–31.6
Height (cm)	172 ± 5	171–173	173 ± 4	171–174	171 ± 6	169–174
Weight (kg)	65.0 ± 4.3	63.9–66.1	64.2 ± 4.0	62.7–65.7	65.8 ± 4.5	64.1–67.5
SP (mmHg)	123.3 ± 12.3	120.1–126.5	123.2 ± 11.9	118.7–127.6	123.5 ± 12.8	118.7–128.3
DP (mmHg)	74.6 ± 9.9	72.1–77.2	74.8 ± 10.0	71.0–78.5	74.5 ± 9.9	70.8–78.2
Hb (g∙dL^−1^)	14.4 ± 0.8	14.2–14.6	14.8 ± 0.8*	14.5–15.0	14.1 ± 0.7	13.8–14.4
Ht (%)	43.9 ± 37.4	43.0–44.8	44.6 ± 4.1	43.1–46.1	43.1 ± 2.6	42.2–44.1

SP, systolic blood pressure; DP, diastolic blood pressure; Hb, hemoglobin level; Ht, hematocrit; *Bold text indicates a statistically significant difference between groups with a *p*-value < 0.05.

### Study Design

The study was conducted between 2015 and 2020. All lab sessions were performed in the last month before the start of league competitions, at the end of the competitors’ preparation period. Motorcycle speedway riders appeared in the laboratory once for blood pressure, hematology, anthropometric parameters, and an assessment of anaerobic capacity with the Wingate test, accompanied by analysis of expired gases and heart rate. All lab sessions were conducted by the same researchers and at the same time of day, in the morning (8:00 a.m.–12:00 a.m.). The subjects were asked to abstain from vigorous exercise, alcohol, and caffeine for 24 h prior to the lab visit.

### Hematological Parameters

Capillary blood was collected from the fingertip of the hand before the exercise test at rest for the assessment of morphotic parameters: hemoglobin level (Hb) and hematocrit (Ht) using an ABX Micros OT.16 (Horiba Medical, Japan).

### Anthropometry and Body Composition

Height and body mass were measured using a WPT 200 medical scale (RADWAG, Poland), whereas body composition was assessed with a FUTREX-6100/XL instrument (Futrex Inc., United Kingdom), based on the near-infrared spectrophotometry method at the center of the biceps of the dominant arm (Fukuda et al., 2017). Each site was measured twice with a caliper and averaged. Percent body fat in total body mass (%FM), fat tissue mass (FM) and lean body mass (LBM) were assessed. Body surface area (BSA) was estimated by applying height and body mass using the equation for men (Yu et al., 2020):

$$\text{BSA }=\;79{\text{.}8106\mathrm{\cdot Height}}^{\text{0}\text{.}7271}{\mathrm{\cdot Weight}}^{\text{0}\text{.}398}$$

### Anaerobic Capacity

The Wingate test (Bar-Or, 1987) was performed to assess anaerobic capacity. The test was performed on an Ergomedic E894 cycloergometer (Monark, Sweden). The cycloergometer was calibrated before the study started. A warm-up was then performed as recommended by the test developers (Bar-Or, 1987). After the warm-up, subjects remained seated on the cycloergometer for 5 min. The flywheel load was 7.5% of the subject’s body mass. The effort lasted 30 s, and the test subject’s task was to work at their maximum rotation rate to reach maximum power as quickly as possible, and to maintain it for as long as possible. The subjects were motivated by shouts to perform as hard as possible. After the test, the subject remained on the cycloergometer for 5 min. The cycloergometer was controlled by computer and MCE v.2.3 software (MCE, Poland). Peak power output (PPO) and total work (TW) were calculated, which were expressed per kilogram of body mass and kilogram of LBM. Fatigue index (FI) was also calculated.

### Cardiorespiratory Responses Analysis

Durint the Wingate test the subjects breathed through a mask, and the expired air was analyzed by a Quark b^2^ device (Cosmed, Milan, Italy). The device was calibrated with atmospheric air and gas mixture of composition: CO_2_—5%, O_2_—16% and N_2_—79% before the measurements began. The recording of respiratory parameters was performed breath by breath. Peak lung ventilation (VE_peak_), oxygen uptake (VO_2peak_), and carbon dioxide excretion (VCO_2peak_) were measured, and the results were averaged every 10 s and converted to minute values to rule out false breaths due to coughing, sighing, and swallowing. Reducing “noise” and artifacts can improve data interpretation. Heart rate (HR) was measured using an RS400 sports-tester (Polar Electro, Finland) during the Wingate test and recorded by Quark b^2^ analyzer software. Again, 10-s averaging was used for HR. The predicted maximum heart rate (HRmax_PRED_) was calculated from the formula (Tanaka et al., 2001):

208-0.7· age,

where: age (years). Based on that, the percent heart rate of contractions (%HR_PRED_) relative to HRmax_PRED_ was calculated.

Systolic and diastolic blood pressure were measured at rest, before and immediately after the Wingate test using an aneroid sphygmomanometer (Riester, Jungingen, Germany). Cardiac stroke volume was calculated using a method that relies on post-exercise blood pressure measurements based on the Jackson (1955) formula:

SV = 101 + (0.5 ·PP) − (0.59· DP) − (0.61 · age),

where: SV–stroke volume (ml), PP–pulse pressure, difference between systolic and diastolic pressure (mmHg), DP–diastolic pressure (mmHg), age (years). The method has been used previously used to verify post-workout changes in VO_2_peak (Hebisz et al., 2016).

### Blood Gasometry and Lactate Concentration

Capillary blood was collected from the fingertip into heparinized capillaries at rest before the start of the test and at the third minute after the end of the test, for determination of acid-base balance: pH, partial pressure of oxygen (pO_2_), partial pressure of carbon dioxide (pCO_2_), and bicarbonate concentration ([HCO_3_^−^]) using a RapidLab 348 analyzer (Bayer, Germany). Based on the blood pH, the hydrogen ion (H^+^) concentration was calculated according to the formula: H^+^ = 10^−pH^. Lactate concentration ([La^−^]) was also measured on a photomer (LP 400 Dr Lange, Germany).

### Sport Level–Total Score per Season

The study used rider classification lists published in the public domain of the organizer of the Polish Speedway Ekstraliga (2021). These consisted of rider results that were obtained during the 2015–2020 seasons analyzed in the paper. The results of the data collection process were numerical sets including the following diagnostic parameters that indicated the riders’ sport levels:

Heats Sum (HS)—The number of heats a rider has participated in during the league season.

Wins Sum (WS)—the number of heats won by the rider.

Points (PTS)—the total number of points scored by a rider in all heats.

Points Sum + Bonus Points (PTS + B)—the total number of points (including bonus points) scored by a competitor in all heats.

Based on these, the following statistics were calculated:

Percentage of wins (%W)—the percentage of heats won (0–100%) by a rider.

Heat Points Average (avPTS)—average number of points scored by a rider per heat (0–3 points).

Heat Points Average Plus Bonus (avPTS + B)—average points (including bonus points) scored by a competitor for one heat (0–3 pts).

Note: a bonus point is awarded to a competitor when rider crosses the finish line directly behind a teammate and ahead of at least one rival. Bonus points are included in the calculation of the average heat or match score. However, they are not included in the team’s total score. The number of matches depends on the league schedule. Eight teams compete in Polish Speedway Ekstraliga, so the regular season consists of fourteen rounds. After this phase is completed, four teams move on to the semifinals, with the winners advancing to the finals. Because of that, the competitors ride during fourteen to eighteen matches per season in the Polish league. In a given match, the number of heats of a rider depends on riders age category. The number of junior heats must be a minimum of two, while the number of senior heats is not regulated, but the maximum number of times a rider starts in one match is seven. In addition, theoretically the competitors with the highest points scored in the match start the last two nominated heats (although in the 14th heat of the 15 heats, the coach has the right to nominate any rider from the team).

### Statistics

IBM SPSS Statistics version 26 software package (IBM, Inc., Chicago, United States) was used to statistically process the data. Results were presented as arithmetic mean ± standard deviation ( $\bar{\text{x}}$ ± SD) and 95% confidence interval (95% CI). Shapiro-Wilk test was used to assess the normality of the distribution of the studied characteristics. Student’s t-test for independent samples was used to assess differences in selected variables between groups. Pearson’s linear correlation coefficient was calculated to examine the relationship between the covariates. The calculated threshold values of correlation coefficients of 0.1, 0.3, 0.5, 0.7, and 0.9 were interpreted as small, moderate, large, very large, and extremely large correlations, respectively (Hopkins et al., 2009). The *p* < 0.05 level was considered statistically significant. Effect size (ES) Cohen’s d was calculated in order to show practical effect, using the following criteria: 0.1—trivial, 0.2—small, 0.5—medium, 0.8—large (Cohen, 1992).

## Results

The junior and senior groups were different in age (*p* < 0.001, *t* = 10.23, ES = 2.64) and blood hemoglobin concentration (*p* < 0.01, *t* = 3.30, ES = 0.85) (Table 1).

The junior group had statistically significantly lower levels of the following anthropometric variables (Table 2):BMI by 4% (*t* = 2.91), %FM by 17.6% (*t* = 3.18), and fat tissue mass by 20.5% (*t* = 3.05). BSA was not different between groups.

**TABLE 2:** Mean ± standard deviation and 95% CI of anthropometrics variables.

Variable	Junior (N = 30)		Senior (N = 30)		Differences between group	
	Mean ± SD	(95% CI)	Mean ± SD	(95% CI)	*p*-value	ES
BMI (kg∙m^−2^)	21.5 ± 1.4*	21.0–22.0	22.4 ± 1.0	22.0–22.8	<0.01	0.75
BSA (m^2^)	1.77 ± 0.06	1.75–1.79	1.78 ± 0.09	1.75–1.81	0.69	0.10
%FM (%)	10.3 ± 2.7*	9.3–11.2	12.5 ± 2.9	11.5–13.6	<0.01	0.79
FM (kg)	6.6 ± 8.3*	5.9–7.4	8.3 ± 2.2	7.5–9.1	<0.01	0.28
LBM (kg)	57.5 ± 3.0	56.4–58.6	57.5 ± 3.8	56.1–59.0	0.99	0

ES, effect size; BMI, body mass index; BSA, body surface area; %FM, percentage of body fat in total body mass; FM, fat mass; LBM, lean body mass. *Bold text indicates a statistically significant difference between groups with a *p*-value < 0.05.

In the aspect of anaerobic capacity tested by the Wingate test, speedway riders had similar levels and differed only in the FI (Table 3). A lower power decrease of 2.3% (*t* = 2.27) was found in the junior group.

**TABLE 3:** Mean ± standard deviation and 95% CI of anaerobic performance determined in the Wingate test.

Variable	Junior (N = 30)		Senior (N = 30)		Differences between group	
	Mean ± SD	(95% CI)	Mean ± SD	(95% CI)	*p*-value	ES
Peak Power (W)	708.7 ± 77.4	679.8–737.6	726.7 ± 63.2	703.1–750.2	0.33	0.25
Peak Power (W∙kg^−1^)	11.0 ± 0.8	10.7 = 11.3	11.0 ± 0.7	10.8–11.3	0.91	0
Peak Power (W∙LBM^−1^)	12.3 ± 1.1	11.9–12.7	12.6 ± 0.9	12.3–13.0	0.19	0.30
Total work (kJ)	16.7 ± 1.4	16.2–17.3	16.8 ± 1.2	16.4–17.3	0.76	0.08
Total work (kJ∙kg^−1^)	260.5 ± 15.7	254.7–266.4	255.9 ± 12.6	251.2–260.7	0.22	0.32
Total work (kJ∙LBM^−1^)	290.7 ± 20.0	283.2–298.1	292.9 ± 16.5	286.7–299.1	0.64	0.12
FI (%)	23.1 ± 4.4*	21.4–24.7	25.4 ± 3.4	24.1–26.6	<0.05	0.58

ES, effect size, *Bold text indicates a statistically significant difference between groups with a *p*-value < 0.05.

Examination of resting levels of VE, VO_2_, VCO_2_ and HR revealed there were not statistically different between the juniors and the seniors riders. In the seniors, VO_2peak_ was higher by 5.8% (*t* = 2.03), while VCO_2peak_ by 12.5% (*t* = 3.51). Peak heart rate was significantly higher in the juniors by 6 (beats ∙ min^−1^) (*t* = 2.05) (Table 4).

**TABLE 4:** Mean ± standard deviation and 95% CI of gas analysis, cardiorespiratory responses, blood gasometry variables and lactate ions concentrations measured at the Wingate test.

Variable	Junior (N = 30)		Senior (N = 30)		Differences between groups	
	Mean ± SD	(95% CI)	Mean ± SD	(95% CI)	*p*-value	ES
VE_peak_ (l∙min^−1^)	121.4 ± 26.1	111.6–131.2	123.7 ± 16.8	117.4–130.0	0.69	0.10
VO_2peak_ (l ∙ min^−1^)	2.7 ± 0.3*	2.6–2.8	2.8 ± 0.3	2.7–3.0	<0.05	0.52
VCO_2peak_ (l ∙ min^−1^)	3.4 ± 0.5*	3.3–3.6	3.9 ± 0.4	3.7–4.0	<0.01	0.91
HR_peak_ (beats ∙ min^−1^)	185 ± 13*	180–190	179 ± 9	176–183	<0.05	0.53
%HR_PRED_ (%)	95.3 ± 6.6	92.8–97.7	95.7 ± 5.5	93.7–97.8	0.76	0.08
SV (ml)	104.1 ± 16.3	98.0–110.2	96.0 ± 23.1	87.4–104.7	0.12	0.40
pH	7.21 ± 0.03	7.20–7.22	7.21 ± 0.05	7.19–7.22	0.87	0
H^+^ (nmol∙L^−1^)	61.9 ± 4.7	60.2–63.7	62.3 ± 6.5	59.9–64.8	0.79	0.07
pO_2_ (mm Hg)	88.0 ± 9.4	84.5–91.5	86.2 ± 13.7	81.1–91.3	0.56	0.15
pCO_2_ (mm Hg)	36.6 ± 6.6	34.1–39.1	36.9 ± 5.9	34.7–39.1	0.84	0.05
[HCO_3_^−^] (mmol∙L^−1^)	14.8 ± 1.6	14.2–15.4	14.8 ± 1.6	14.2–15.4	0.99	0
[La^−^] (mmol∙L^−1^)	12.1 ± 2.0	11.3–12.8	11.7 ± 2.1	11.0–12.5	0.54	0.16

ES, effect size, VE_peak_–peak minute ventilation, VO_2peak_ - peak oxygen uptake, VCO_2peak_–peak carbon dioxide exertion, HR_peak_–peak heart rate, %HR_PRED_, percentage of predicted heart rate; SV, stroke volume, H^+^ - hydrogen ions concentration, pO_2_—oxygen partial pressure, pCO_2_—carbon dioxide partial pressure, [HCO_3_^−^]—bicarbonate ions concentration, [La^−^]—lactate ions concentration. * Bold text indicates a statistically significant difference between groups with a *p*-value < 0.05.

Resting level of oxygen partial pressure was significantly different between groups (*p* < 0.01, *t* = 2.69, ES = 0.69), which were 66.6 ± 5.8 (95% CI 64.4–68.8 mm Hg) and 71.5 ± 8.0 (95% CI 68.5–74.5 mm Hg) in the juniors and seniors, respectively. Blood lactate concentration at rest was lower in the senior group (*p* < 0.05, *t* = 2.06, ES = 0.53) reaching 0.8 ± 0.3 (95% CI 0.7–1.0 mmol∙L^–1^), while in the junior group was 1.1 ± 0.5 (95% CI 0.9–1.2 mmol∙L^–1^). Both were within the resting physiological normal range. Blood gas level parameters and lactate concentration measured after the Wingate test were not statistically different between the test groups of riders (Table 4).

The both groups were not statistically different in terms of the number of heats per season. The seniors had 37.8% more wins (*t* = 2.56) and had a 35.0% higher winning percentage (*t* = 3.07) compared to the juniors. The number of points scored by the juniors was lower by 26.3% (*t* = 2.49), and including bonus points, the difference was 24.5% (*t* = 2.42). The seniors had a higher average of points (*t* = 3.47) and points including bonuses (*t* = 3.49) earned per heat (Table 5).

**TABLE 5:** Mean ± standard deviation and 95% CI of the sport levels indicators of the riders, as determined by the scores obtained during all heats in the league season.

Variable	Junior (N = 30)		Senior (N = 30)		Differences between group	
	Mean ± SD	(95% CI)	Mean ± SD	(95% CI)	*p*-value	ES
HS (n)	65.2 ± 18.1	58.4–72.0	71.2 ± 16.5	65.1–77.4	0.18	0.35
WS (n)	13.3 ± 9.7*	9.7–17.0	21.4 ± 14.3	16.1–26.7	<0.05	0.66
%W	18.6 ± 10.6*	14.6–22.5	28.6 ± 14.3	23.2–33.9	<0.01	0.79
PTS (n)	88.3 ± 47.2*	70.6–105.9	119.8 ± 50.8	100.8–138.7	<0.05	0.64
avPTS (0–3)	1.3 ± 0.4*	1.1–1.4	1.6 ± 0.4	1.5–1.8	<0.001	0.90
PTS + B (n)	99.8 ± 51.8*	80.4–119.6	132.2 ± 51.7	112.9–151.5	<0.05	0.63
avPTS + B (0–3)	1.4 ± 0.4*	1.3–1.6	1.8 ± 0.4	1.7–1.9	<0.001	0.90

ES, effect size; HS, heats sum; WS, wins sum; %W, percent of wins; PTS, points; avPTS, heat points average; PTS + B**-** points sum + bonus points, avPTS + B- heat points average plus bonus. *Bold text indicates a statistically significant difference between groups with a *p*-value < 0.05.

We found numerous statistically significant correlations between sport levels indices and anthropometric or anaerobic capacity parameters in the senior group, and when calculated considering all participants. Only age correlated with wins sum (r = −0.37, *p* < 0.05) and percentage of wins (r = −0.36, *p* < 0.05) in the juniors. Among the seniors, body height correlated with wins sum (r = −0.44, *p* < 0.05), percentage of wins (r = −0.52, *p* < 0.01), points sum (r = −0.41, *p* < 0.05), points sum + bonus points (r = −0.41, *p* < 0.05), avPTS (r = −0.53, *p* < 0.01), avPTS + B (r = −0.55, *p* < 0.01). Also in this group of riders, LBM negatively correlated with percentage of wins (r = −0.41, *p* < 0.05), avPTS (r = −0.39, *p* < 0.05), avPTS + B (r = −0.42, *p* < 0.05). In addition, BSA in seniors was inversely related to wins sum (r = −0.40, *p* < 0.05), percentage of wins (r = −0.48, *p* < 0.01), points sum + bonus points (r = −0.37, *p* < 0.05), avPTS (r = −0.45, *p* < 0.01) and avPTS + B (r = −0.48, *p* < 0.01). However, PPO correlated with percentage of wins (r = −0.39, *p* < 0.05), avPTS (r = −0.41, *p* < 0.05) and avPTS + B (r = −0.41, *p* < 0.05). Blood Hb level correlated with heats sum (r = 0.38, *p* < 0.05) and points sum + bonus points (r = −0.37, *p* < 0.05). Similarly, pO_2_ measured after the Wingate test correlated with r = 0.44 (*p* < 0.05) and r = 0.38 (*p* < 0.05) with heats sum and points sum + bonus points, respectively. Post-exercise CO_2_ partial pressure was inversely related to wins sum (r = −0.38, *p* < 0.05), percentage of wins (r = −0.49, *p* < 0.01), avPTS (r = −0.45, *p* < 0.05) and avPTS + B (r = −0.47, *p* < 0.01). H^+^ ion concentration correlated with avPTS (r = −0.38, *p* < 0.05).

**FIGURE 1:**
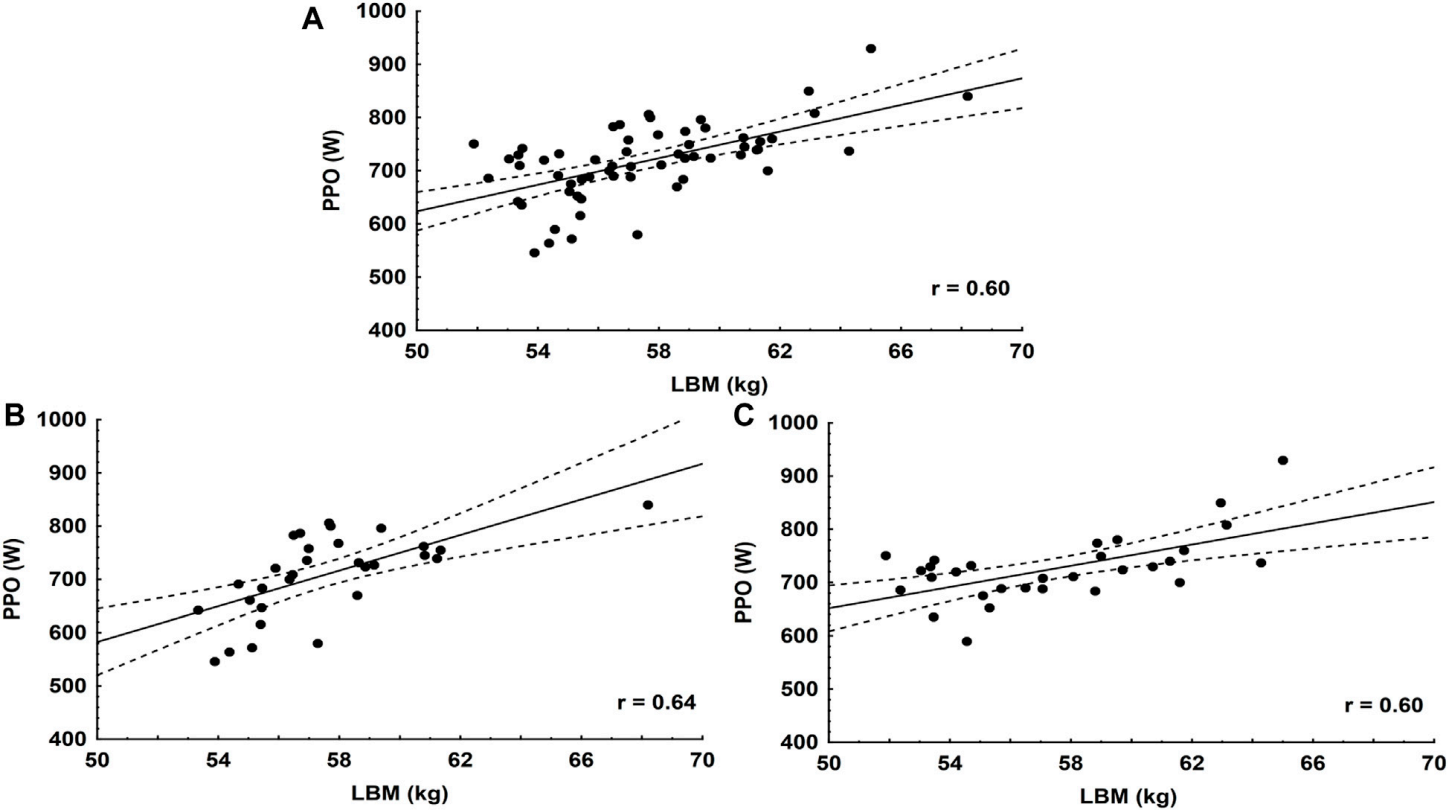
Associations between LBM and PPO in: All speedway riders **(A)**, juniors **(B)**, seniors **(C)**.

Only LBM and PPO, as well as BSA and PPO, showed significant relationships with selected indicators related to the sport level of the speedway riders. Thus, we correlated these variables with each other. Significant correlations could be found (*p* < 0.01) between LBM and PPO in the juniors, the seniors and all speedway riders (Figure 1). BSA correlated with PPO in the juniors at r = 0.71 (*p* < 0.001), while at r = 0.75 (*p* < 0.001) in seniors, and at r = 0.70, (*p* < 0.001) in all speedway riders.

## Discussion

The present study compared body composition components, anaerobic capacity, acute cardiorespiratory and biochemical responses on the Wingate test, as well as sport levels of elite motorcycle speedway riders in junior and senior age categories. Our main findings were: 1) seniors present a higher sport level and score more points, despite the lack of significant differences in the number of heats completed during the season; 2) lean body mass does not differ between groups, but juniors have a lower percentage of body fat and lower fat tissue mass; 3) seniors achieve a higher level of oxygen consumption and carbon dioxide excretion, and a greater decrease in power in the Wingate test; 4) body height correlated negatively with all parameters characterizing the sport levels of motorcycle speedway riders; 5) lean body mass, body surface area, hydrogen ion concentration, and carbon dioxide partial pressure measured after maximal anaerobic exercise show an inverse relationship with the average number of points scored per season.

The breakdown by age category that is provided in this research is based on the official speedway league rules. A comparison of the two groups is lacking in the literature. We chose to compare these two age groups in order to provide a point of reference for researchers, coaches, and the riders themselves. Motorcycle speedway racing is extremely popular in Europe, including Poland, and riders who are 16 years of age or older can participate in the league competitions. The mean age of the riders we studied was similar to those published in a recent article on motorcycle speedway racing by Martin et al. (2019), who reported a mean age of 24.4 ± 5.3 (years). However, the foregoing authors evaluated the sport levels of the riders using a different scoring method (Calculating Match Average) than the one presented in this paper. Furthermore, Martin et al. (2019) did not divide competitors by age, unlike the current study’s methodology. Having considered that, this paper presents a novel approach in this aspect.

The current results indicate that seniors exhibit a higher sport level, score at more heats, have higher winning percentage and scoring averages, despite no difference in total heats during the season. This may demonstrate the importance of increasing experience with successive years of training and competitions. Martin et al. (2019) sought to explain differences in sport levels by evaluating anthropometric data and using specific tests to assess motor and movement abilities. Although their study had an age difference of 2.6 years and training experience of 1.3 years, the assumed level of statistical significance was not found (Martin et al., 2019). Because we used a different approach in diagnosing the fitness and performance of the speedway subjects, we are not able to directly compare the results obtained in this area with the results of our own study. Therefore, we will refer to the recorded and presented values of anthropometric indicators, which are commonly used when studying motor sports representatives.

It is widely accepted that motorsport competitors should be characterized by adequate height and body mass (Sánchez-Muñoz et al., 2011), as the higher power-to-weight ratio of the motorcycle + rider system allows for greater acceleration and top speed (Martin et al., 2019). Since speedway motorcycles are restricted by speedway sport regulations as to minimum weight (77 kg), a lighter and smaller rider is desirable to exploit the potential of the generated engine power (D'Artibale et al., 2008; Sánchez-Muñoz et al., 2011; Martin et al., 2019). The anthropometric data presented indicate that among elite speedway riders, seniors were similar to juniors in terms of height, body mass, and lean body mass. This allows us to conclude that these anthropometric characteristics do not change in subsequent years of training and should be taken into account at the stage of rider recruitment and selection. In addition, we assessed body fat levels in total body mass. We have shown that the groups we studied differ in this regard, and that seniors achieve higher scores, although being characterized by a higher fat content and mass. These values are within the normal range of the athletic population, but specific nutritional interventions could be implemented to prevent significant fluctuations in fat tissue mass. The speedway riders we studied (including seniors and juniors) had similar body heights and lower body mass, as well as BMIs, compared to those representing the British Premier League, with heights at 171.2 ± 6.6 (cm), body mass at 69.7 (kg) and BMIs at 23.4 (kg∙m^−2^) (Martin et al., 2019). The aforementioned authors also observed that high performers are slightly heavier than low performers, which has not been confirmed by the present research. Findings from this study are comparable to those of representatives from other motorcycle sports, where riders’ body size has been considered as a trait affecting final performance (Gobbi et al., 2005; Sánchez-Muñoz et al., 2011; Bach et al., 2015). This is supported by reported statistically significant negative correlations (moderate to high) between body height, body mass, BSA, LBM, and score indices (WS, %W, PTS, avPTS, PTS + B, avPTS + B). This provides an important rationale for coaches in recruiting riders to the speedway racing, as coaches should pay special attention to body height, which was the only parameter that correlated with all scoring indicators.

Speedway riders compete for about 60 s during heats, therefore we tested their anaerobic capacity with the commonly used Wingate test for lower extremities. We observed that the study groups did not differ in relative and absolute power levels and work performed. Only the seniors had a larger drop in maximum power than the juniors, which did not change the fact that the measured minimum power was virtually identical. The reference values for peak power achieved by the Wingate test performed by National Collegiate Athletic Association (NCAA) Division IA male competitive riders classify the speedway riders we studied with 11.0 W kg^−1^ as a group with low anaerobic capacity (Coppin et al., 2012). The lack of differences between juniors and seniors allows us to conclude that it is likely that the anaerobic potential of seniors is developed to an insufficient extent in subsequent years of training. The implication is that riders have reserves in this aspect, and the application of proper training should raise anaerobic potential to a higher level. According to our knowledge, this is the first study to analyze elite speedway riders with the Wingate test and document their anaerobic capacity. When referring to representatives of other motorsports, Bach et al. (2015) reported higher anaerobic power levels in highly trained MX riders 940.4 ± 86.1 (W) and 12.7 ± 0.8 (W∙kg^−1^). The differences may be due to the different testing methodology and the applied resistance of 8.4% body mass vs. 7.5% in the current study. Ascensão et al. (2008) applied the Wingate test to the upper extremities, investigating the physiological, biochemical and functional changes induced during a simulated 30-min ride in a warm environment for MX riders. Nevertheless, it is well known that peak power output values for the upper extremities are lower compared to the lower extremities (Price et al., 2014). Therefore, subsequent studies of speedway riders could include the use of the Wingate test for upper extremities.

Surprisingly, the current study showed that excessively high maximum power is negatively correlated to the winning percentage and average point scores of seniors and when all competitors were included. The maximum power generated in the lab test is dependent on lean body mass, which is consistent with many other studies from various sports. The relationship between body composition and anaerobic capacity has been reported in a number of sports, where the level of these traits is crucial for high performance, e.g., wrestlers (Vardar et al., 2007), ice hockey players (Potteiger et al., 2010) and rowers (Durkalec-Michalski et al., 2019). In the context of motorcycle speedway sport, having high lean mass translates into maximum power generated in the Wingate test, but both LBM and PPO negatively correlate with the sport levels of the competitors expressed by the percentage of heats won in a season and the average points scored (including bonus points). Therefore, it seems that there must be a trade-off between body size and muscle-generated power. Further comprehensive studies should clarify this issue and identify potential limits on the height and weight, BSA, or optimal LBM and %FM levels of motorcycle speedway riders. Such information should be useful to riders and coaches to increase power and muscle efficiency. The importance of which in speedway racing was reported by Martin et al. (2019), they avoided gaining lean body mass.

Analysis of acute cardiorespiratory and biochemical responses assessed in and after the Wingate test also provided interesting information. Seniors achieved higher VO_2_peak and VCO_2_peak compared to juniors. Although HRpeak was lower in seniors, relative values to predicted maximum value did not differ, just like other hemodynamic indices. Unfortunately, we cannot relate this to maximal values and determine aerobic input, because we did not perform an incremental exercise test like researchers diagnosing VO_2_peak of highly trained Motocross riders (Bach et al., 2015) or off-road motorcyclists (Gobbi et al., 2005). We only assume that the riders used similar muscle mass due to the lack of differences in LBM. Due to the lack of scientific reports, such a study, e.g., with the RAMP test (Michalik et al., 2019), would increase the existing state of knowledge of aerobic capacity in speedway riders. At the same time, there were no significant differences in post-exercise blood lactate, hydrogen ion and bicarbonate concentrations. This indicates a greater efficiency of energy production by the aerobic system in seniors (Price et al., 2014), which makes a significant contribution during 60 s of intense speedway rider effort (Gastin, 2001). Admittedly, it should be taken into account that we diagnosed these responses during the Wingate laboratory test and not in field conditions at the speedway track. Subsequent studies should address the shortcomings of these scientific evidences. Only in the senior group significant correlations were found between post-effort hydrogen ion concentration, blood CO_2_ partial pressure, and average points scored per heat. This demonstrates the importance of metabolic acidosis tolerance capacity, buffer capacity, and respiratory compensation (McKenna et al., 1997), which are important for delaying the onset of fatigue and the ability to maintain high muscle work intensity (Wan et al., 2017). This indicates the need for intensive training, e.g. with the interval method, also in the junior age category. Repetitive sprint training could be reasonable, due to riders performing intense anaerobic activities every dozen/several dozen minutes or so during a speedway match.

This study contributes to the level of knowledge available in the literature about motorcycle speedway sport, but some limitations should be considered. Further studies should include performing body composition assessment using the commonly used dual-energy X-ray absorptiometry (DXA). One should also consider the Wingate test designed to evaluate the upper extremities and the use of upper and lower limb flexor and extensor force moments. Further research is needed to determine the physiological, biochemical and hormonal responses of the body during a simulated heat under track conditions. It also seems interesting to evaluate changes in riders’ physique and body composition, anaerobic capacity levels before and after the competitive season. Longitudinal studies evaluating the effectiveness of training interventions to improve driving performance are warranted. Such observations are necessary to familiarize coaches and riders with training practices to better understand the functional characteristics of the elite speedway rider’s body, and to implement more effective training interventions.

## Conclusion

Senior speedway riders are characterized by a higher sport level than juniors, which seems to be due to the experience acquired in the subsequent years of training, as well as due to the greater contribution of aerobic energy during maximal effort. The riders’ sport level is influenced by anthropometric parameters, especially body height, body surface area and lean body mass, hence the crucial consideration of these indices in the recruitment of riders to speedway sport. Development of anaerobic power should occur without an accompanying increase in body mass, especially lean body mass. The importance of tolerance of increasing hydrogen ion concentrations and efficient removal of carbon dioxide from the body indicate the need for completing high-intensity interval training sessions.

## Data Availability

The raw data supporting the conclusion of this article will be made available by the authors, without undue reservation.
